# The Foreign Journals: Animal Chemistry and Pharmacy

**Published:** 1842-01

**Authors:** 


### ANIMAL CHEMISTRY AND PHARMACY.

*On the State in which Urea exists in the Urine.* By MM. Cass and Henry.

The authors have previously asserted (Journal de Pharmacie, tom. xxiii,) that urea exists in the urine in combination with lactic acid, and perhaps also with phosphoric acid, and this assertion having been questioned by M. Lecanu, MM. Cass and Henry have made new experiments to prove the existence of normal lactate of urea. Fresh urine was evaporated to five sixths of its volume, at a heat below 120°; and the brown acid liquor which remained was filtered and concentrated at a gentle heat towards the consistence of syrup, then dried *in vacuo.* The dry residuum was agitated in a flask with ten or twelve times its weight of a mixture of two parts of sulphuric æther and one part of rectified alcohol. After some days of contact and agitation the æthereal liquor was re-filtered and saturated by a slight excess of alkaline carbonate. A new filtration was effected, and the liquor exposed to a very gentle heat gave beautiful prismatic crystals of lactate of urea, identical with those obtained by the direct combination of urea and lactic acid.

*Journal de Pharmacie. Juin,* 1841.

---

*On the Nutritive Qualities of Gelatine.*

Ten years ago a commission was appointed by the Institute of France to enquire into the properties of gelatine as a material for the food of the poor. The members were MM. Thenard, D'Arcet, Dumas, Flourens, Serres, Breschet, and Magendie (reporter), and the question which they set as the scope of their enquiries was, " Is it possible by any economic plan to extract from bones an aliment which, either alone or mixed with other substances, may take the place of meat?" The report now presented in answer is very lengthened; the conclusions are as follows:

1. It is not possible, by any known plan, to extract from bones an aliment, which, either alone or mixed with other substances, can take the place of meat. 2. Gelatine, albumen, fibrine, taken separately, nourish animals for only a very limited time, and in a very imperfect manner. In general they soon excite an insurmountable disgust, so that the animals prefer to die rather than touch them. 3. These same proximate principles, artificially united and made agreeable to the taste by seasoning, are taken more readily and longer than where they are given separately; but in the end they have no better influence on nutrition, for the animals, though they take them in considerable quantities, at last die with all the signs of complete inanition. 4. Muscular flesh, in which gelatine, albumen, and fibrine are united according to the laws of organic nature, and are associated with other substances such as fat, salts, &c., is sufficient, even in very small quantity for complete and prolonged nutrition. 5. Raw bones have the same advantage, but the quantity consumed in twenty-four hours must be much greater than that of meat which will suffice. 6. Every kind of preparation, such as boiling in water, the action of muriatic acid, and especially the transformation into gelatine, diminishes the nutritive qualities of bones, and even seems in some cases to destroy them altogether. 7. The commission, however, does not wish to pronounce at once on the employment of gelatine associated with other aliments in the nourishment of man, knowing that only direct experiments can throw a clear light on that subject; they are now actively occupied with such experiments, and the results of them will be explained in the second and last part of the report. 8. Gluten, such as is extracted from wheat-flour, is sufficient of itself for complete and prolonged nutrition. 9. Fatty substances, taken alone for food, support life for some time, but they give rise to an imperfect and disordered nutrition in which fat accumulates in all the tissues, sometimes in the form of oleine and stearine, sometimes in that of almost pure stearine. [We have given only the conclusions of this report, because dogs having been the subjects of the experiments the results, though thoroughly established as far as those animals are concerned, are only probably applicable in the physiology of man. They abundantly confirm the generally admitted truth of the insufficiency of proximate principles for food; but the main question, namely, to what extent bone-soup is a good article to form a part of human diet, and how it may be best prepared, remains unanswered.]

*Archives Générales de la Médecine. Sept.* 1841.

---

*On the Preservation of Ferruginous Pills.* By M. Simonin, of Nancy.

The following method is said to have the effect of preserving ferruginous pills in an unalterable state, and maintain their proper consistence, which is not obtained by the ordinary formula:

Take pure protosulphate of iron,
pure subcarbonate of potash, of each equal parts.

Reduce separately into a fine powder, then mix and triturate together until they begin to liquefy. Add a sufficient quantity of clarified honey to render the mixture completely liquid. Place the mortar on a very slow fire, and constantly triturate until the mass assumes a pilular consistence. Preserve in a pot or divide into pills.

*Bulletin Général de Thérapeutique. Janvier* 15 *et* 30, 1841.

